# Associations of Depression and Social Isolation Risk Among Adults Age 60 and Older

**DOI:** 10.1093/geroni/igaa057.1019

**Published:** 2020-12-16

**Authors:** Matthew Smith, Lesley Steinman, Matthew Barrett, Leigh Ann Eagle, Sue Lachenmayr, Basia Belza

**Affiliations:** 1 Texas A&M University, College Station, Texas, United States; 2 University of Washington, Seattle, Washington, United States; 3 MAC, Salisbury, Maryland, United States

## Abstract

Background. Depression and social isolation are believed to be strongly interrelated. Social isolation can lead to depression because of reduced human contact and connectivity. Depression can cause withdrawal from interpersonal encounters and fuel feelings of social isolation. Despite causality, this study aimed to examine the relationship between depression and social isolation risk among older adults. Methods. Using an internet-delivered survey, data were analyzed from a national sample of 4,082 adults age 60 years and older. The survey intended to validate the Upstream Social Isolation Risk Screener (U-SIRS), a 13-item screener (Cronbach’s alpha=0.80) to assesses physical, emotional, and social support aspects of social isolation. Theta scores for the U-SIRS served as the primary independent variable, which were generated using Item Response Theory. Depression was the dependent variable for this study, which was identified using the PHQ-2 (scores of 3+ indicated risk for depression). Binary logistic regression was used to identify factors associated with depression. Results. Participants’ average age was 69.6(±5.2) years, 59% of participants were female, and 9% met depression criterion. Depressive symptomology and U-SIRS theta scores were positively significantly correlated (r=0.56, P<0.001). Participants with higher U-SIRS theta scores (OR=3.52, P<0.001), with more chronic conditions (OR=1.16, P<0.001), and without people they felt close to and could call for help (OR=1.76, P=0.004) were more likely to report depression. Conclusion. Given the strong interrelation of depression and social isolation risk, coordinated efforts are needed to both treat depressive symptomology and link older adults to resources and services that facilitate meaningful interactions with others.

